# The “SALPARE study” of spontaneous intracerebral hemorrhage: part 1

**DOI:** 10.1186/s42466-023-00231-1

**Published:** 2023-02-02

**Authors:** Ludovica De Rosa, Renzo Manara, Francesca Vodret, Caterina Kulyk, Florian Montano, Alessio Pieroni, Federica Viaro, Maria Luisa Zedde, Rosa Napoletano, Mario Ermani, Claudio Baracchini

**Affiliations:** 1 Stroke Unit and Neurosonology Laboratory, Padua University Hospital, Padua, Italy; 2 Neuroradiology Unit, Padua University Hospital, Padua, Italy; 3 Stroke Unit and Neurosonology Laboratory, Department of Neurology, Johannes Kepler University Linz, Linz, Austria; 4 Neuroradiology, Department of Medicine and Surgery, University of Salerno, Salerno, Italy; 5 Neurology Unit, Stroke Unit, AUSL-IRCCS di Reggio Emilia, Reggio Emilia, Italy; 6 UOC Neurologia AOU S. Giovanni di Dio e Ruggi d’Aragona, Salerno, Italy; 7 Service of Medical Statistics, Department of Neurology, Padua University Hospital, Padua, Italy

**Keywords:** Cerebral hemorrhage, Stroke, Hematoma, Anticoagulants, Predictors

## Abstract

**Background:**

Spontaneous intracerebral hemorrhage (ICH) is a devastating type of stroke with a huge impact on patients and families. Expanded use of oral anticoagulants and ageing population might contribute to an epidemiological change. In view of these trends, we planned a study to obtain a contemporary picture and identify early prognostic factors to improve secondary prevention.

**Methods:**

This multicenter prospective cohort study included consecutive adult patients with non-traumatic ICH admitted to three academic Italian hospitals (Salerno, Padova, Reggio Emilia) over a 2-year period. Demographic characteristics, vascular risk profile, clinical data and main radiological characteristics were correlated to 90-day clinical outcome.

**Results:**

Out of 682 patients [mean age: 73 ± 14 years; 316 (46.3%) females] enrolled in this study, 40% died [86/180 (47.8%) in Salerno, 120/320 (37.5%) in Padova, 67/182 (36.8%) in Reggio Emilia; *p* < 0.05)] and 36% were severely disabled at 90 days. Several factors were associated with a higher risk of poor functional outcome such as antithrombotic drug use, hyperglycemia, previous cerebrovascular accident, low platelet count, and pontine/massive/intraventricular hemorrhage. However, at multivariate analysis only pre-ICH mRS score (OR 30.84), GCS score at presentation (OR 11.88), initial hematoma volume (OR 29.71), and NIHSS score at presentation (OR 25.89) were independent predictors of death and poor functional outcome.

**Conclusion:**

Despite the heterogeneity among centers, this study on ICH has identified four simple prognostic factors that can independently predict patients outcome, stratify their risk, and guide their management.

**Supplementary Information:**

The online version contains supplementary material available at 10.1186/s42466-023-00231-1.

## Introduction

For the past 30 years, spontaneous intracerebral hemorrhage (ICH) has been overshadowed by a generalized interest of the neurological community on the new strategies to treat ischemic stroke [1]. Yet, ICH carries a devastating social burden due to its high mortality and disability rates [2]. In fact, a significant percentage of patients die in the short term, while survivors have a considerable residual disability, and are at risk of serious neurological complications such as epilepsy and cognitive impairment. In contrast to ischemic stroke, ICH has also fewer treatment options, and this might induce a nihilistic approach and a less organized care with negative consequences on prognosis [3]. Furthermore, the growing indications for use of direct oral anticoagulants, coupled with an aging population, suggest that the number of anticoagulated patients will continue to expand, and this might have a negative impact on the epidemiology of ICH [4].

The current situation requires a collection of epidemiological data and the recognition of outcome predictors. Therefore we decided to conduct a multicenter prospective study in three academic centers representing Northern, Central and Southern Italy, in order to: (i) explore the pathophysiology of ICH, (ii) calculate mortality and disability rates at 90 days from onset, and (iii) identify demographic, clinical and radiological characteristics that might serve as predictors of 90-day functional outcome.

## Patients and methods

### Study population

This prospective multicenter cohort study included consecutive adult (≥ 18 years) patients with non-traumatic ICH admitted to three Italian University Hospitals (Padova, Reggio Emilia and Salerno) over a 2-year period (from January 1st 2016 to December 31st 2017). Patients with a spontaneous ICH confirmed by a head CT at hospital admission were eligible for enrollment. Exclusion criteria were: traumatic intracranial bleeding, subdural hemorrhage, primary subarachnoid hemorrhage, primary intraventricular hemorrhage, hemorrhagic conversion of ischemic stroke, ICH associated with thrombolytic treatment for ischemic stroke, cerebral hemorrhage secondary to brain tumor or vascular malformation or vasculitis or venous thrombosis.

### Data collection

Demographic characteristics, vascular risk profile, clinical data and radiological characteristics were recorded. In particular, collected data included age, sex, family history of ICH, history of cerebrovascular/cardiovascular events, hypertension, diabetes mellitus, chronic obstructive pulmonary disease, liver failure, chronic kidney disease, antiplatelet or anticoagulant therapy, smoking, alcohol and illicit substance consumption.

Patients were diagnosed as having arterial hypertension, if they had a systolic blood pressure ≥ 140 mmHg or diastolic blood pressure ≥ 90 mmHg or if they were or had been on antihypertensive medication at any time before enrollment. Diabetes mellitus was defined as fasting serum glucose ≥ 7.0 mmol/L (≥ 126 mg/dl), non-fasting serum glucose ≥ 11.1 mmol/L (≥ 200 mg/dl), glycated hemoglobin (HbA1C) ≥ 48 mmol/mol (≥ 6.5% by the Diabetes Control and Complications Trial) or the use of glucose-lowering drugs before enrollment. History of previous cerebrovascular events was defined as any history of transient ischemic attack, ischemic stroke or hemorrhagic stroke. History of previous cardiovascular events was defined as any history of angina pectoris, myocardial infarction, coronary treatment (angioplasty/stenting, or bypass surgery). Patients were diagnosed as having chronic kidney disease, if they had a GFR < 60 ml/min/1.73 m^2^ for ≥ 3 months. The diagnosis of chronic obstructive pulmonary disease was based on an obstructive spirometry (FEV1/FVC < 0.7, assessed prebronchodilator). Patients with liver failure were defined as patients who had been diagnosed with chronic hepatitis or liver cirrhosis prior to ICH, or who showed abnormal laboratory data for aspartate aminotransferase (> 50 IU/l), alanine aminotransferase (> 50 IU/l), or gamma-glutamyl transferase (> 60 IU/l). Smoking habit was defined as current or cessation within the past 5 years. Alcohol consumption was defined as current consumption of > 1 drink per day for women, > 2 drinks per day for men, or cessation within the past 5 years. Illicit substance consumption was defined as current consumption of illicit/controlled (cocaine, heroin, marijuana, benzodiazepines, and methadone) substances or cessation within the past 5 years.

The clinical picture of all patients was assessed by the Glasgow Coma Scale (GCS) score and by the National Institute of Health Stroke Scale (NIHSS) on hospital admission, at 24–48 h and at hospital discharge. The degree of disability before stroke was based on an anamnestic modified Rankin Scale (mRS 0–5), while functional outcome at 90 days was assessed at the 3-month follow-up visit (mRS 0–5) or anamnestically for the deceased patients (mRS 6).

Concerning baseline cerebral CT scan images, the following characteristics were recorded: ICH location (eg. deep, lobar, infratentorial), hematoma volume, intraventricular extension. The hematoma volume was calculated according to the ABC/2 method, where A is the greatest hemorrhage diameter, B is the diameter 90 degrees to A, and C is the approximate number of CT slices with hemorrhage multiplied by the slice thickness [5, 6]. In patients with follow-up CT performed within 48 h from baseline, hematoma expansion (relative hematoma volume growth > 33% or absolute hematoma volume growth > 6 mL) [7] and the time interval between first and second CT were also recorded.

The independent committee for the evaluation of the CT scans was composed by 3 radiologists: A.S. in Salerno, R.P. in Reggio Emilia, and F.C. in Padova. Initial and follow-up CT images, early CTA (within 6 h from onset) and cerebral MRI images were centrally evaluated by R.M., a neuroradiologist with more than 20-years experience in cerebrovascular diseases and blind to the patient’s clinical picture.

Data collection was approved by the Ethics Committee of each hospital and informed consent was obtained from participants or their family members.

Patients were treated medically or surgically at the physician’s discretion, according to the standard of care of each recruiting center. Patients who died or underwent intracranial surgery before follow-up CT scan were excluded from the evaluation and the analysis on hematoma expansion.

### Statistical analysis

Continuous data are reported as mean ± standard deviation (SD) and are compared by Student's t-test, while categorical variables are reported as proportions and compared by Mann–Whitney *U-*test or χ2 test as appropriate.

Potential predictors of poor outcome known from the literature were included as candidate variables. Clinical outcome at 90 days was categorized as favorable (mRS ≤ 2) and poor (mRS ≥ 3). The test characteristics (odds ratio, sensitivity, specificity and associated 95% confidence intervals) were calculated directly for disconnected variables or after dichotomization (Youden method) for ordinal and normal variables. Multivariate analysis was performed using the ordinal logistic regression model to identify the independent predictors of functional outcome after three months. The ordinal variable was obtained by stratifying the outcome as class 0 (mRS 0–2), class 1 (mRS 3–5) and class 2 (mRS 6). The predictive power of the model was then estimated by using the first-year cohort as the instruction set and the second year cohort as the validation set. A *p* < 0.05 was considered significant.

All statistical analyses were performed using SPSS Version 26 e STATISTICA Version 13.

## Results

### Descriptive analysis

Between January 1st 2016, and December 31st 2017, 682 consecutive patients with spontaneous ICH were recruited across three centers: University hospital of Padova, 320 patients (163 females); University hospital of Salerno, 180 patients (76 females); and University hospital of Reggio Emilia, 182 patients (77 females). Demographics, baseline risk factor characteristics, and clinical data of patients with ICH are summarized in Table 1. Mean age was 73 ± 14 years, and 54% of patients were male. There were significant differences among recruiting centers, with regards to smoking, previous cardiovascular events, arterial hypertension, chronic obstructive pulmonary disease, current treatment with non-vitamin K antagonists or single antiplatelet regimen, pre-ICH mRS score, and severity of clinical syndrome.

**Table 1:** Demographics, baseline risk factors, and clinical data of recruited patients with ICH

		All centers (n = 682)	Padova (n = 320)	Salerno (n = 180)	Reggio Emilia (n = 182)	*p*
Age (years, mean ± SD)		73 ± 14	73 ± 14	72 ± 13	75 ± 13	NS
Sex	Male, *n* (%)	366 (54%)	157 (49%)	104 (58%)	105 (58%)	NS
Family history of ICH	Yes, *n* (%)	11 (2%)	9 (3%)	2 (1%)	0	NS
Smoking	Yes, *n* (%)	185 (27%)	77 (24%)	30 (17%)*	78 (43%)*	< 0.01
History of previous cerebrovascular events	Yes, *n* (%)	141 (20%)	78 (24%)	30 (16%)	33 (18%)	NS
Type of previous cerebrovascular	TIA	14 (10%)	9 (11%)	4 (13%)	1 (3%)	NS
	Ischemic stroke	79 (56%)	37 (48%)	18 (60%)	24 (73%)	NS
	Hemorragic stroke	43 (30%)	28 (36%)	8 (27%)	7 (21%)	NS
	Both	5 (4%)	4 (5%)	0	1 (3%)	NS
History of previous cardiovascular events	Yes, *n* (%)	115 (17%)	39 (12%)*	29 (16%)	47 (26%)*	< 0.01
Diabetes mellitus	Yes, *n* (%)	128 (19%)	65 (20%)	36 (20%)	27 (15%)	NS
Alcohol abuse (< 5 years)	Yes, *n* (%)	50 (7%)	25 (8%)	7 (4%)	18 (10%)	NS
Drug abuse (< 5 years)	Yes, *n* (%)	6 (1%)	4 (1%)	0	2 (1%)	NS
Hypertension	Yes, *n* (%)	464 (68%)	243 (76%)*	108 (60%)*	113 (62%)	< 0.01
Chronic obstructive pulmonary disease	Yes, *n* (%)	43 (6%)	17 (5%)	22 (12%)*	4 (2%)*	< 0.01
Liver failure	Yes, *n* (%)	35 (5%)	24 (7%)	6 (3%)	5 (3%)	NS
Anticoagulant therapy	VKA, *n* (%)	74 (11%)	40 (12.5%)	17 (10%)	21 (12%)	NS
	Non-VKA, *n* (%)	53 (8%)	34 (11%)*	15 (8%)	4 (2%)*	< 0.01
Antiplatelet therapy	Single, *n* (%)	194 (28%)	104 (32%)*	36 (20%)*	56 (31%)	< 0.01
	Dual, *n* (%)	11 (1.6%)	14 (4%)	0	1 (0.5%)	NS
Blood glucose levels > 180 mg/dl, *n* (%)		116 (17%)	63 (20%)	28 (16%)	24 (13%)	NS
INR > 1.7, *n* (%)		77 (11%)	37 (12%)	21 (12%)	19 (10%)	NS
Platelet < 100*10^3^/mm^3^, *n* (%)		31 (5%)	14 (4%)	10 (5%)	7 (4%)	NS
mRS pre-stroke	0–2	474 (69%)	241 (75%)*	79 (44%)*	151 (83%)*	< 0.01
	3–5	98 (14%)	73 (23%)*	4 (2%)*	23 (12%)*	< 0.01
GCS at onset	≤ 8, *n* (%)	130 (19%)	69 (22%)*	25 (14%)*	36 (20%)	NS
	9–13, *n* (%)	120 (18%)	68 (21%)*	15 (8%)*	37 (20%)	< 0.01
	> 13, *n* (%)	306 (45%)	174 (54%)	32 (18%)*	100 (55%)*	< 0.01
NIHSS at onset, *median* (range)		10 (0–40)	10 (0–40)	5 (0–25)*	18 (1–28)*	< 0.01

Asterisk denotes values that significantly differ from each other

*ICH* intracerebral hemorrhage, *TIA* transitory ischemic attack, *INR* International Normalized Ratio, *mRS* modified Rankin Scale, *GCS* Glasgow Coma Scale, *NIHSS* National Institute of Health Stroke Scale

### Neuroradiological features

A total of 727 ICH were detected by cerebral CT, namely 336 in Padova, 194 in Salerno and 197 in Reggio Emilia. Baseline radiological cohort characteristics are summarized in Additional file 1: Table S1. Among 727 ICH, 348 (47.8%) were located in the cerebral lobes, 201 (27.1%) in the striatal/capsular area, 107 (14.7%) in the thalamus and thalamic-capsular area, 42 (5.8%) in the cerebellum and 28 (3.9%) in the brainstem. Mean hematoma volume was 40.6 ± 53.1 ml, while its median and interquartile range were 17.6 ml and 4.5–53.7 ml, respectively. After correction for multiple comparisons, there were no significant differences regarding hematoma location, volume and features among recruiting centers. Multiple and concomitant ICH were found in 23 (3.3%) patients (3 in Padova, 10 in Salerno and 10 in Reggio Emilia). During the 2-year recruitment period, 22 (3%) patients suffered from spatially distinct intracerebral hemorrhages (i.e. recurrent hemorrhages: 13 in Padova, 4 in Salerno and 5 in Reggio Emilia).

Acute CTA was performed only in 105 out of 682 patients (15%) with a significant difference among centers: 2/182 (1%) in Reggio Emilia, 76/320 (24%) in Padova and 27/180 (15%) in Salerno. The spot sign, defined as at least one focus of contrast extravasation within the hematoma, not connected with any surrounding blood vessels and having any size or shape and density ≥ 120 Hounsfield units, was found in 27 (25,7%) patients (see Additional file 1: Table S2).

Follow-up CT was obtained in 556 patients (79%), with no significant differences among recruiting centers. However, time-to-follow-up CT differed significantly (31 ± 25 h in Padova, 64 ± 61 h in Salerno and 51 ± 77 h in Reggio Emilia; *p* < 0.001). Control CT was performed within 24 h in 388 patients (55%), namely 225 (67%) in Padua, 64 (35%) in Salerno, and 99 (53%) in Reggio Emilia (*p* < 0.001).

Hematoma expansion (164/556 patients, 29.5%) and intraventricular bleeding (246/556 patients, 44.2%) did not differ among the three study groups (see Additional file 1: Table S3).

Cerebral MRI was performed in 230/682 (34%) patients, with a significant difference among centers: 82/182 (45%) in Reggio Emilia, 116/320 (36%) in Padova and 32/180 (18%) in Salerno.

### Clinical course

**Fig. 1:**
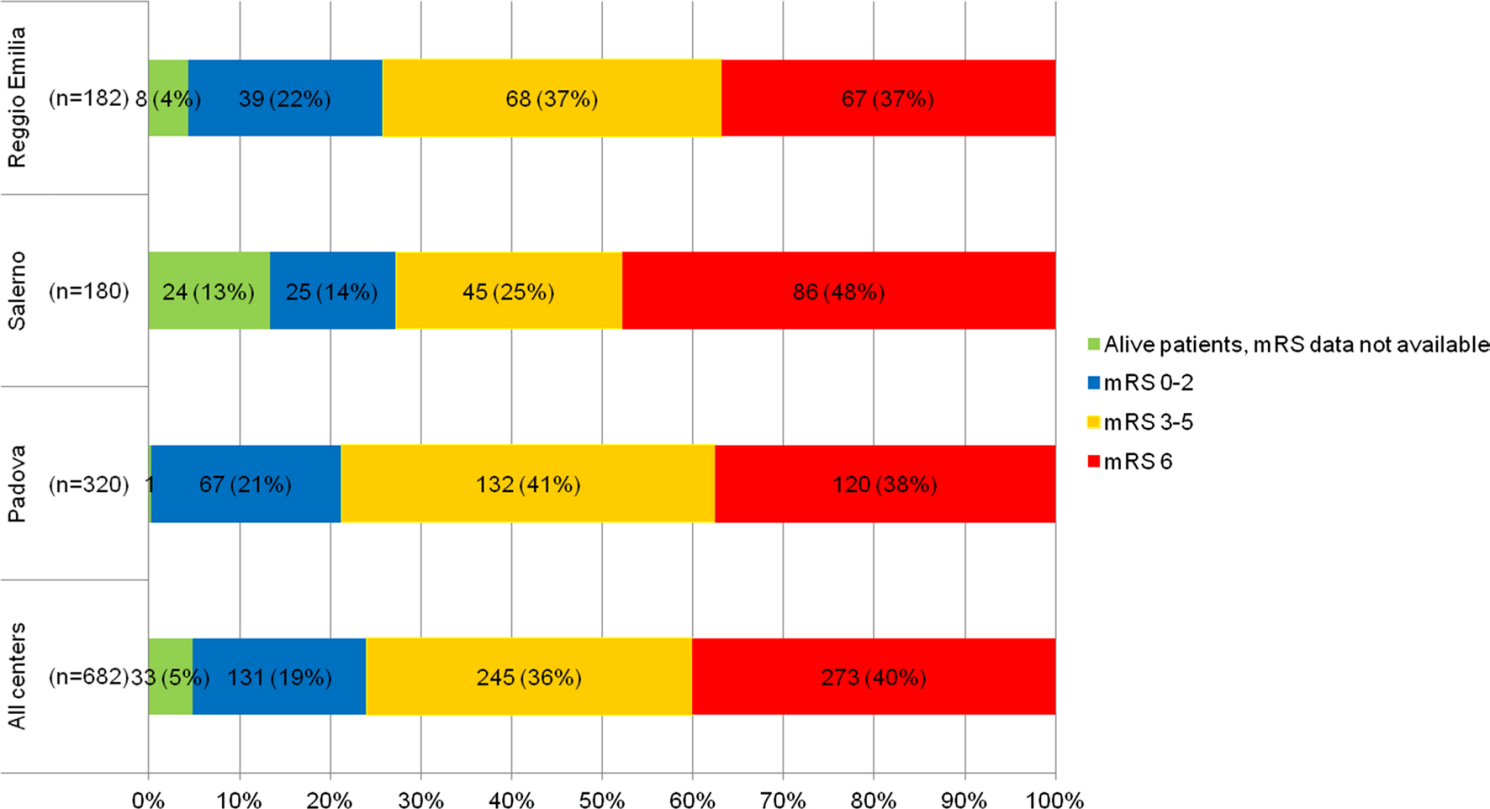
Mortality and disability at 90 days from the index event

At 90 days from the event, 273/682 patients (40%) had died and there was a significant difference in mortality among centers [86/180 (47.8%) in Salerno, 120/320 (37.5%) in Padova, 67/182 (36.8%) in Reggio Emilia; *p* < 0.05)]. Instead the disability rate was similar (Table 2; Fig. 1).

**Table 2:** Mortality and disability at 90 days from the index event

3-month mRS	All centers (n = 682)	Padova (n = 320)	Salerno (n = 180)	Reggio Emilia (n = 182)
0–2, *n* (%)	131 (19%)	67 (21%)	25 (14%)	39 (22%)
3–5, *n* (%)	245 (36%)	132 (41%)	45 (25%)	68 (37%)
6, *n* (%)	273 (40%)	120 (38%)	86 (48%)	67 (37%)
Survivors, mRS data not available	33 (5%)	1 (0.3%)	24 (13%)	8 (4%)

**Fig. 2:**
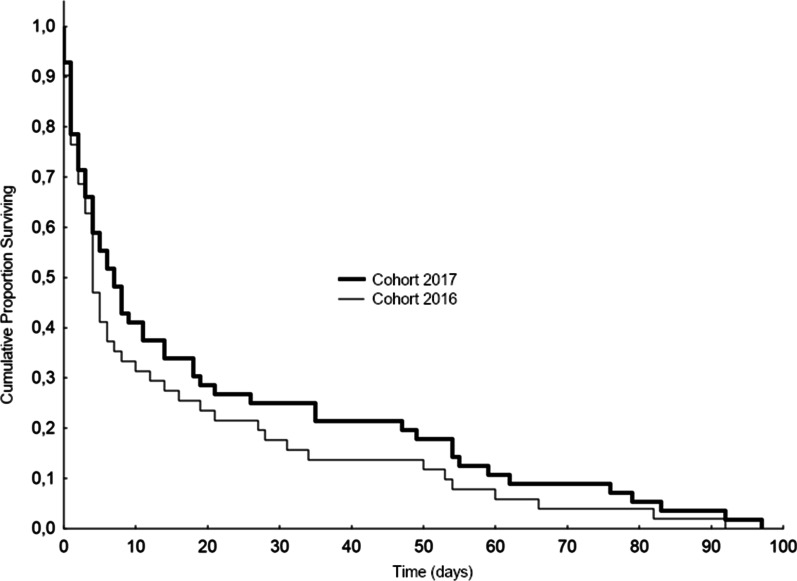
Survival rates (2016 vs. 2017) in the Padua cohort among deceased patients at 3 months

In the Padua cohort, three-month mortality rates (39.8% vs. 32.18%; *p* = 0.17) did not differ significantly between the first and the second year of study. Death occurred mostly within 30 days: 50% within 5 days, 75% within 21 days (Fig. 2).

### Predictors of outcome

Patients with the following characteristics had a significantly (*p* < 0.05) higher risk of poor functional outcome (mRS > 2): higher premorbid modified Rankin Scale score (≥ 1), previous cerebrovascular accident, anticoagulant and/or antiplatelet therapy at admission, higher blood glucose levels (> 180 mg/dl), lower platelet count (< 100 000/mm^3^), lower GCS score at onset (≤ 14), higher NIHSS score at onset (≥ 9), pontine and massive hemorrhage, intraventricular bleeding, larger baseline hematoma volume (> 15 mL), and hematoma expansion (Table 3).

**Table 3:** Univariate and multivariate regression analyses for predictors of poor outcome (mRS > 2)

	Univariate		Multivariate	
Variables	*P* value	OR	*P* value	OR
Premorbid mRS ≥ 1	0.00000	4.88 (2.82–8.44)	0.00000	30.84
Previous cerebrovascular accident	0.00659	2.12 (1.22–3.71)		
Ongoing anticoagulant therapy	0.05106	1.71 (0.99–2.96)		
Ongoing antiplatelet therapy	0.00654	1.86 (1.18–2.93)		
Blood glucose level > 180 mg/dl	0.00016	3.48 (1.76–6.90)		
Platelet count < 100/mm^3^	0.01093	8.72 (1.17–64.62)		
GCS score at the onset ≤ 14	0.00000	8.92 (5.46–14.58)	0.00000	11.88
NIHSS score at the onset ≥ 9	0.00000	8.93 (5.43–14.67)	0.001	25.89
Pontine or massive hemorrhage	0.01374	1.24 (1.09–2.75)		
Intraventricular bleeding	0.00000	3.94 (2.52–6.16)		
Baseline hematoma volume > 15 ml	0.00000	6.67 (4.15–10.72)	0.00000	29.71
Hematoma expansion (i.e. > 30%)	0.00087	2.42 (1.42–4.11)		

**Fig. 3:**
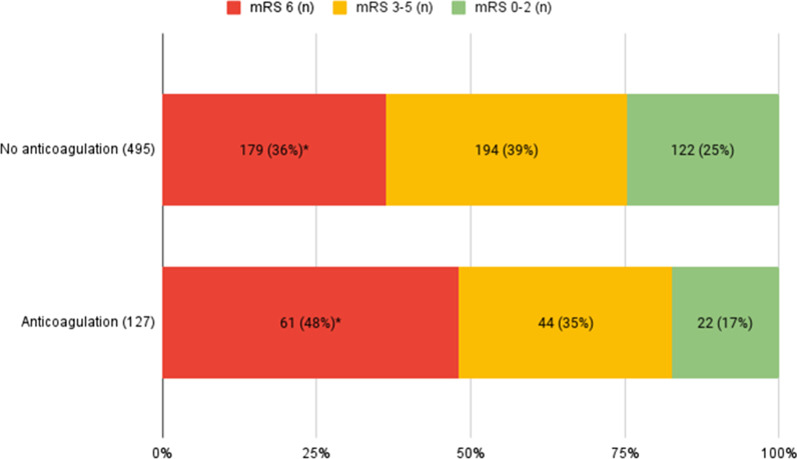
Three-month mortality and disability rates in anticoagulated versus non-anticoagulated patients, *p = 0.01

Predictors for death at 90 days are reported in Additional file 1: Table S4. At admission, 127/682 patients (19%) were on anticoagulants. Anticoagulation therapy was associated with a higher three-month mortality rate (*p* < 0.05) (Fig. 3).

In the multivariate analysis, only the following parameters were found to be independently associated with a poor outcome (*p* < 0.05): initial hematoma volume, GCS score at onset, NIHSS score at onset and pre-ICH mRS score (Table 3).

In this 2-year study, we used the first-year cohort as the instruction set and the second year cohort as the validation set in order to derive an ordinal logistic function *g*_c_, which assigns a probability *p*_*c*_ to each category *c* = {1, 2, 3}, where 1 means mRS = 0–2, 2 means mRS = 3–5, and 3 means mRS = 6. The probability *p*_*c*_ is given by: *p*_*1*_ = *g*_*1*_; *p*_*2*_ = *g*_*2*_*-g*_*1*_; *p*_*3*_ = *1−p*_*1*_*-p*_*2*_, where *g*_c_ with *c* = {1, 2} is calculated as follows:

$$g_{c} = 1/\left[ {1 + e^{{\left( { - a_{c} + \mathop \sum \limits_{i}^{N} \beta_{i} x_{i} } \right)}} } \right]$$

where: *a*_*c*_ is the *c*-dependent intercept “1, 2” (*a*_*1*_ = −2,128; *a*_*2*_ = 0,493); *x*_*i*_ is the independent variable, with *i*_*1*_ = baseline hematoma volume, *i*_*2*_ = mRS pre-stroke, *i*_*3*_ = baseline GCS, *i*_*4*_ = baseline NIHSS; and *ß*_*i*_ are the corresponding weights. The signs of *ß* parameters are in agreement with the clinical interpretation of the *x*_*i*_ variables (see Additional file 1: Table S5). The above model has a predictive power of 73% for class I (mRS 3–5) and 64% for class II (mRS 6).

## Discussion

This Italian multicenter prospective study shows that: (1) the 3-month outcome is still poor in patients suffering from brain intraparenchymal hemorrhage even though mortality and disability rates are slightly better than previously reported; (2) outcome differences among centers are evident and likely reflect a different management in the acute phase; (3) clinical and imaging data at onset remain strong predictors of outcome in spite of the likely improved treatments; (4) early phases of disease are most crucial for survival and research should focus on this period to prevent re-bleeding and mass effect.

Previous population studies and traditional registry data showed that ICH is a devastating condition with high mortality and disability rates. Specifically, during the past decades almost half of patients were reported dead within the first 30 days [8–11]. Compared to that scenario, our study shows a slight improvement of patients’ survival, in line with recent studies [12, 13]. Fernando and colleagues assessed mortality rate in patients with spontaneous ICH in the entire adult population of Ontario, Canada, over a ten-year period (April 1, 2009–March 30, 2019). Similarly to our data, they found a 30-day mortality rate of 34.7%, and a 1-year mortality rate of 45.4%. Over the study period, both short- and long-term mortality decreased, indicating a continuous improvement of neurocritical care [13]. Other recent reports [14, 15] have suggested a reduction in short-term mortality of ICH patients, although these studies are small and represent institutional cohorts that might not depict a “real-life” scenario of ICH. Broad inclusion criteria as applied in our study show that every five patients with spontaneous ICH, two die, two remain severely disabled and only one regains functional independence.

However, outcome differences among centers still exist, most likely reflecting a different management in the acute phase and suggesting that further therapeutic improvement is possible. Specifically, we observed a worse prognosis when follow-up CT was delayed or ancillary exams such as CT-angiography or MRI were less frequently performed. These aspects likely represent a marker of a less aggressive global approach in some centers, revealing an undermining nihilism which might impact negatively on prognosis. Recent studies have shown that a rapid implementation of evidence-based, guideline-recommended care to acute ICH patients is significantly associated with a lower 30-day case fatality [16], suggesting that an active approach is deemed necessary. In fact, monitoring these patients in neurointensive care units or in semi-intensive stroke units leads to better clinical outcomes [17]. In this context, the identification of early reliable outcome predictors might aid in the selection of ICH patients benefiting significantly from a more intensive care.

In the present study, simple clinical and imaging characteristics at onset (GCS/NIHSS/mRS scores, previous cerebrovascular accident, anticoagulation, antiplatelet therapy, hyperglycemia, lower platelet count, baseline hematoma volume, IVH, hematoma expansion, pontine and massive ICH) were significant predictors of outcome in ICH patients. Yet, only GCS/NIHSS at admission, pre-ICH mRS scores and baseline hematoma volume were found to be independent predictors of outcome at multivariate analysis.

Unexpectedly, anticoagulant use at time of admission was not recognized as such, in line with a recent report [18]. There seems not to be a clear explanation for this finding. In our study, anticoagulation therapy was not associated with hematoma volume at onset, hematoma expansion rate or early clinical deterioration, but only with pre-ICH mRS score, a surrogate marker for concomitant comorbidities which might explain our results.

As to the independent early predictors, pre-ICH disability, larger hematoma and severe clinical syndrome point to a worse prognosis, as expected. Their detection might be useful for identifying subgroups of patients to be directed towards a more aggressive management and possibly new treatments as their natural course would otherwise be fatal. In future trials, the prediction model we propose might help assess and compare treatment effects on different study populations.

Recent studies [19–22] have investigated the prognostic role of early neuroradiological features of the cerebral hematoma. These will be extensively addressed in part 2 of this study.

Future research should focus on the very early phases of intracranial bleeding, as survival analyses from our data and from previous studies [13, 23] show that mortality rate is highest in the early period after ICH, with three quarters of deaths occurring within the first three weeks. It is clear that very early interventions are necessary to decrease mortality and severe disability rates which at the moment represent nearly four fifths of patients suffering from ICH.

This is an observational study, therefore it does not have the benefit of randomization to allocate by chance risk factors for the outcome of interest, that is 90-day functional outcome. Moreover: 1. Time from onset to first CT was not pre-specified. In many patients this data was unavailable, as the precise time of onset was unknown (i.e. insidious onset of the symptoms, wake-up ICH, coma as presenting symptom). However, the exclusion of patients with unclear symptom onset from the analysis would have resulted in a selection bias (eg. excluding patients presenting with coma would mean excluding those with a worse prognosis). 2. Pre-ICH treatment was heterogeneous. However, according to our results both antiplatelet agents and anticoagulants were not independent predictors of poor outcome. 3. No pre-specified diagnostic work-up and acute therapy were indicated; the decision was left to the treating physician. 4. Decision to surgery was taken on a clinical basis and according to the CT at onset (no patient underwent surgery after the follow-up CT). Reasons for surgical intervention were the same for all three centers: superficial hemorrhage; clot volume between 20 and 80 ml; worsening neurological status; hemorrhage causing midline shift/raised ICP.

Given these limitations, one should remember that much medical knowledge and current practice still rests on a foundation of observational research, which will continue to have an important role in providing the information needed to improve medical decision-making.

## Conclusions

Heterogeneity in diagnostic workup and prognosis among study centers indicates that there is still ample space for improving ICH care. Risk stratification by means of reliable and easily measured predictors of outcome, as found in our study, is necessary for guiding future research towards a better care of ICH patients.

## Supplementary Information

**Additional file 1. Table S1:** Baseline radiological cohort characteristics. **Table S2:** CTA findings. **Table S3:** Follow-up CT scan timing and findings. **Table S4:** Univariate analysis for predictors of mortality. **Table S5:** Independent predictors outcome at 90 days.

## Data Availability

All data generated or analysed during this study are included in this published article [and its Additional file 1].
