# Supplementary material for: The “SALPARE study” of spontaneous intracerebral hemorrhage: part 1
Source: Neurol Res Pract. 2023 Feb 2;5:5. doi: 10.1186/s42466-023-00231-1 (PMC9893659; doi:10.1186/s42466-023-00231-1)
Supplement: Supplementary file 1 — Additional file 1. Table S1: Baseline radiological cohort characteristics. Table S2: CTA findings. Table S3: Follow-up CT scan timing and findings. Table S4: Univariate analysis for predictors of mortality. Table S5: Independent predictors outcome at 90 days. [file 42466_2023_231_MOESM1_ESM.docx]

**SUPPLEMENTARY MATERIAL**

**Table 1: Baseline radiological cohort characteristics.**

|  | | | All centers  (n=727) | Padova  (n=336) | Salerno  (n=194) | Reggio Emilia  (n=197) |
| --- | --- | --- | --- | --- | --- | --- |
| Location | Cerebral lobes | | 348 (48%) | 163 (48.5%) | 85 (44%) | 100 (51%) |
|  | Striato-capsular area | Anterior | 25 (3.4%) | 12 (3.6%) | 7 (3.6%) | 6 (3.1%) |
|  |  | Middle | 7 (1%) | 5 (1.5%) | 2 (1%) | 0 |
|  |  | Lateral | 40 (5.5%) | 17 (5.1%) | 9 (4.6%) | 14 (7.2%) |
|  |  | Posteromedial | 8 (1.1%) | 1 (0.3%) | 4 (2.1%) | 3 (1.6%) |
|  |  | Posterolateral | 56 (7.7%) | 29 (8.6%) | 13 (6.7%) | 14 (7.2%) |
|  |  | Massive | 65 (8.9%) | 26 (7.7%) | 26 (13.4%) | 13 (6.7%) |
|  | Thalamocapsular area | | 59 (8%) | 30 (8.9%) | 14 (7.2%) | 15 (7.7%) |
|  | Thalamus | | 48 (6.6%) | 22 (6.6%) | 13 (6.7%) | 13 (6.7%) |
|  | Cerebellum | | 42 (5.8%) | 25 (7.4%) | 10 (5.1%) | 7 (3.6%) |
|  | Pons | | 24 (3.3%) | 5 (1.5%) | 9 (4.6%) | 10 (5.1%) |
|  | Midbrain | | 4 (0.6%) | 1 (0.3%) | 1 (0.5%) | 2 (0.1%) |
|  | Undetectable | | 1 (0.1%) | 0 | 1 (0.5%) | 0 |
| Volume [ml, mean±SD, median (IQR)] | | | 40.6±53.1  17.6 (4.5-53.7) | 39.9±53.5  18.1 (6.0-52.9) | 39.4±51.6  17.8 (3.5-54.5) | 41.6±53.9  16.9 (3.4-54.2) |
| Intraventricular bleeding | | | 335 (46%) | 166 (49.4%) | 86 (44.3%) | 83 (42.1%) |

**Table 2: CTA findings.**

|  | All centers  (n=105, 15%) | Padova  (n=76, 24%) | Salerno  (n=27, 15%) | Reggio Emilia  (n=2, 1%) |
| --- | --- | --- | --- | --- |
| NCCT-to-CTA time, min | 146.7 | 158.9 | 102.4 | 240 |
| Spot sign, *n* | 27 | 20 | 5 | 2 |
| Other findings |  | 8 AVM;  3 fistulas;  2 aneurysm;  2 venous thrombosis;  1 Moyamoya disease. | 1 AVM;  1 aneurysm. | 1 cavernous angioma. |

NCCT: non-contrast CT; AVM: artery-venous malformation.

**Table 3: Follow-up CT scan timing and findings.**

|  | All centers  (n=556) | Padova  (n=261) | Salerno  (n=145) | Reggio Emilia  (n=150) |
| --- | --- | --- | --- | --- |
| Time between the initial and follow-up CT scan (hours, mean±SD) | 45±55 | 31±25 | 64±61 | 51±77 |
| ICH Volume (ml, mean±SD) | 30.54±40.7 | 31.70±42.9 | 31.12±40.9 | 28.7±30.9 |
| Intraventricular bleeding | 246 (44.2%) | 119 (45.6%) | 67 (46.2%) | 60 (40%) |
| Hematoma expansion | 164 (29.5%) | 70 (26.8%) | 45 (31%) | 49 (32.7%) |

**Table 4: Univariate analysis for predictors of mortality.**

| Variables | P value | OR |
| --- | --- | --- |
| mRS pre-stroke ≥ 1 | 0.00000 | 2.65 (1.82-3.84) |
| Previous cerebrovascular accident | 0.00259 | 1.83 (1.23-2.74) |
| Previous cardiovascular event | 0.00676 | 1.78 (1.17-2.72) |
| Diabetes mellitus | 0.02542 | 1.58 (1.05-2.37) |
| COPD | 0.02285 | 2.05 (1.09-3.87) |
| Liver failure | 0.02926 | 2.14 (1.06-4.30) |
| Chronic kidney disease | 0.00006 | 3.13 (1.75-5.57) |
| Ongoing anticoagulant therapy | 0.00831 | 1.72 (1.14-2.58) |
| Ongoing antiplatelet therapy | 0.00181 | 1.74 (1.22-2.47) |
| Blood glucose level >180 mg/dl | 0.00000 | 2.93 (1.93-4.46) |
| Platelet count <100/mm^3^ | 0.00341 | 2.95 (1.38-6.28) |
| GCS at the onset ≤ 13 | 0.00000 | 28.39 (17.11-47.13) |
| NIHSS score at the onset ≥19 | 0.00000 | 10.44 (6.55-16.64) |
| Intraventricular bleeding | 0.00000 | 3.13 (2.25-4.37) |
| Baseline hematoma volume > 30 ml | 0.00000 | 6.40 (4.49-9.14) |
| Hematoma expansion | 0.00201 | 1.92 (1.26-2.93) |

**Table 5: Independent predictors outcome at 90 days.**

|  | Weights *ß_i_* | SE | *p* |
| --- | --- | --- | --- |
| Baseline hematoma volume >15 mm^3^ | ,017 (,011; ,024) | ,003 | ,000 |
| mRS pre stroke | ,451 (,292; ,610) | ,081 | ,000 |
| Baseline GCS | -,167 (-.251; -0,84) | ,043 | ,000 |
| Baseline NIHSS | ,042 (,018; ,066) | ,012 | ,001 |
